# Navigating Without a Map: Unpacking Pedagogical Viewpoints of Health Sciences Educators Without Educational Training

**DOI:** 10.1007/s40670-025-02528-z

**Published:** 2025-10-10

**Authors:** Gabriel Hervas, José Luis Medina

**Affiliations:** Department of Didactics and Educational Organization, University of Barcelona, Barcelona, Spain

**Keywords:** Qualitative research, Pedagogical viewpoints, Teaching methods, Teacher role, Student role

## Abstract

**Introduction:**

An essential attribute of competent health sciences educators (HSEs) is the integration of disciplinary and pedagogical knowledge. However, many HSEs receive little or no formal educational training, raising concerns about their teaching approaches. This study addresses this issue by examining the perceptions of HSEs without formal pedagogical training regarding two central aspects: teaching and learning methods and the roles they assign to themselves and their students.

**Methods:**

The research employs a qualitative phenomenological case study involving nine HSEs from diverse fields—medical education, nursing, and podiatry—who collaborated in the observation and discussion of their own teaching practices. Discussions held around these observations were inductively analyzed to identify interpretive dimensions related to the participants’ conceptualizations of their own roles, their students’ roles, and their use of teaching and learning methods.

**Results:**

The findings indicate that, although participants valued methods beyond traditional lectures and acknowledged the importance of aligning teaching with assessment, they simultaneously upheld a lecture format where students primarily act as passive listeners and attributed to external factors—senior faculty or students—the difficulties in implementing more participatory strategies. Regarding their own role, participants emphasized sharing professional experience and serving as role models over content delivery. They perceived their responsibility for student motivation and success in the course as limited, approaching success through grades rather than learning. In terms of the student role, participants expected learners to assume responsibility for their own learning, demonstrated through independent study and in-class participation, distinguishing this from a focus on passing a course and grades.

**Discussion:**

The study reveals that while HSEs without educational training may express views aligned with educational literature, their interpretations often lack depth, remain teacher-centered, overlook pedagogical nuances, externalize difficulties, and exhibit unacknowledged inconsistencies. These findings highlight the importance of formal educational development programs to foster reflective practice and pedagogical growth among HSEs.

## Introduction

We could say that the ultimate goal for health sciences educators (HSEs) is ensuring that students come to think like, perform like, and perceive themselves as health sciences professionals [1]. Fulfilling such a necessary—and ambitious—goal related to students’ cognitions, behaviors, and feelings is within the grasp of competent HSEs who, among other essential attributes, should be knowledgeable about education theory and capable of engaging in education scholarship [2].

However, among the roles of academic HSEs, that of educator is the least recognized, to the point that previous studies [3–5] report its peripheral relevance and that academics who commit themselves to teaching over research or clinical practice endanger their credibility, status, prestige, and incomes. Thus, HSEs frequently consider themselves clinicians and researchers rather than educators and are often under pressure to yield clinical outputs [6]. This is a common trait in higher education, where teachers tend to pay less attention to teaching practice and to their professional development as educators and more attention to areas that offer more recognition and more attractive career paths [7]. This pattern continues to occur despite relevant international pedagogical movements promoting an inquiry approach to teaching-learning similar to that of research (see, for example, the Scholarship of Teaching and Learning) [8, 9].

Previous studies show that educational development programs contribute to improving HSEs’ perceptions of the value of teaching [10] and that they are more effective when they consist of master’s degrees in education rather than short courses [2]. However, in many contexts, HSEs (or university teachers, overall) do not need to undergo any kind of mandatory training to teach, leaving it to each faculty member to decide on their own whether to participate in educational development programs. In this way, health sciences education has escaped the thorough examination and quality control that exist for clinical practice in the health sciences [7]. As a result, a high percentage of HSEs, even when they take up important educational roles at their institutions, receive little or no training in the theory of education, teaching strategies, or assessment [7, 11, 12], which in turn might affect their identity as educators [13].

These circumstances raise concerns about the pedagogical approach of HSEs, for whom the concepts of pedagogy and didactics might be largely absent [14]. Thus, in this study, we aimed to answer the following research question: What are the perceptions that HSEs without pedagogical training have about two key elements of their educational practice (teaching and learning methods, and the role of teachers and learners in the teaching and learning process)?

## Method

### Study Design and Sample

Given our research goal of uncovering HSEs’ perceptions, we performed a qualitative phenomenological case study, an inquiry approach that is useful, also in research in medical education [15], for accessing the meanings that the participants ascribe to experiences and phenomena and for developing inductive interpretations that remain sensitive to the context [16, 17]. In conducting our research, we were mindful of the call by Steinert et al. [18] for more qualitative studies of initiatives related to the professional development of HSEs.

The study was conducted at the School of Medicine and Health Sciences of the author’s institution, a public university ranked among the top 75 in the 2025 Times Higher Education Top Medical School Ranking, where it received ethical approval (Institutional Review Board 00003099). Participants were nine HSEs teaching in the undergraduate degrees of medicine, nursing, and podiatry, as members of the departments of Clinical Sciences (*n* = 4, 44.4%) and Medical-Surgical and Basic Nursing (*n* = 5, 55.6%) and with diverse teaching experience: under 5 years (*n* = 2, 22.2%), between 5 and 10 years (*n* = 2, 22.2%), from 10 to 20 years (*n* = 3, 33.3%), and over 20 years (*n* = 2, 22.2%). Participants collaborated in observing and discussing three different lessons taught to a total of 140 students and were selected among a population of 95 faculty members of the School who expressed their interest in improving and innovating in their courses for meeting three criteria: (1) willingness to engage in a process known as lesson study [19, 20] that involved cooperating to design a lesson, observing its implementation, and meeting in a post-lesson discussion to talk about how the design and the delivery worked out; (2) lecturers from the different disciplines present at the School (only Dentistry had no representation in the population), under the premise that interprofessional collaboration should model educational development initiatives [21], especially in the context of the health sciences, where it is recognized as a critical factor in enhancing health care [22]; (3) agreed to be observed and audio- and video-recorded during the whole process.

### Data Collection

To identify the perceptions of HSEs, we used as a primary data source the recordings of the discussions that the participants carried out after each of the lessons was delivered. Using these as the main data source emerges from a sociocultural and sociocognitive approach to discourse [23, 24], through which we understand HSEs’ professional talk as the visible display of their thinking and as a reflection of their priorities and their knowledge and ideas about a topic. To gain a better comprehension of the participants’ discourse and insights during these discussions, we observed the delivery of the lessons and conducted individual pre- and post-semistructured interviews. Interviews aimed to help us understand the discussion based on the participants’ professional biographies and accurately interpret the data by encouraging the participants to describe their reasoning [25] so that we could have access to their thought processes [26] during the recorded discussions, monitoring the alignment between what they said and what they reported that they had said [27].

### Data Analysis

We transcribed the discussions, amounting to 5 h17 min34 s, and conducted content analysis [28] to identify, quantify, and interpret the data related to participants’ perspectives on teaching and learning methods and the different roles of educators and students. This was done phrase by phrase, microanalytically, following an inductive and iterative process of selection, categorization, and constant comparisons based on the procedures of grounded theory [29]. The authors conducted open and axial coding of the three transcripts to inductively identify the themes that appeared, refining their properties, accuracy, and sensitivity to ensure reliability after each post-lesson discussion was analyzed. During this process, the conversations were divided into segments based on theme. Segment length varied from 3 s to 4 min46 s but mostly remained under 60 s.

## Results

**Fig. 1:**
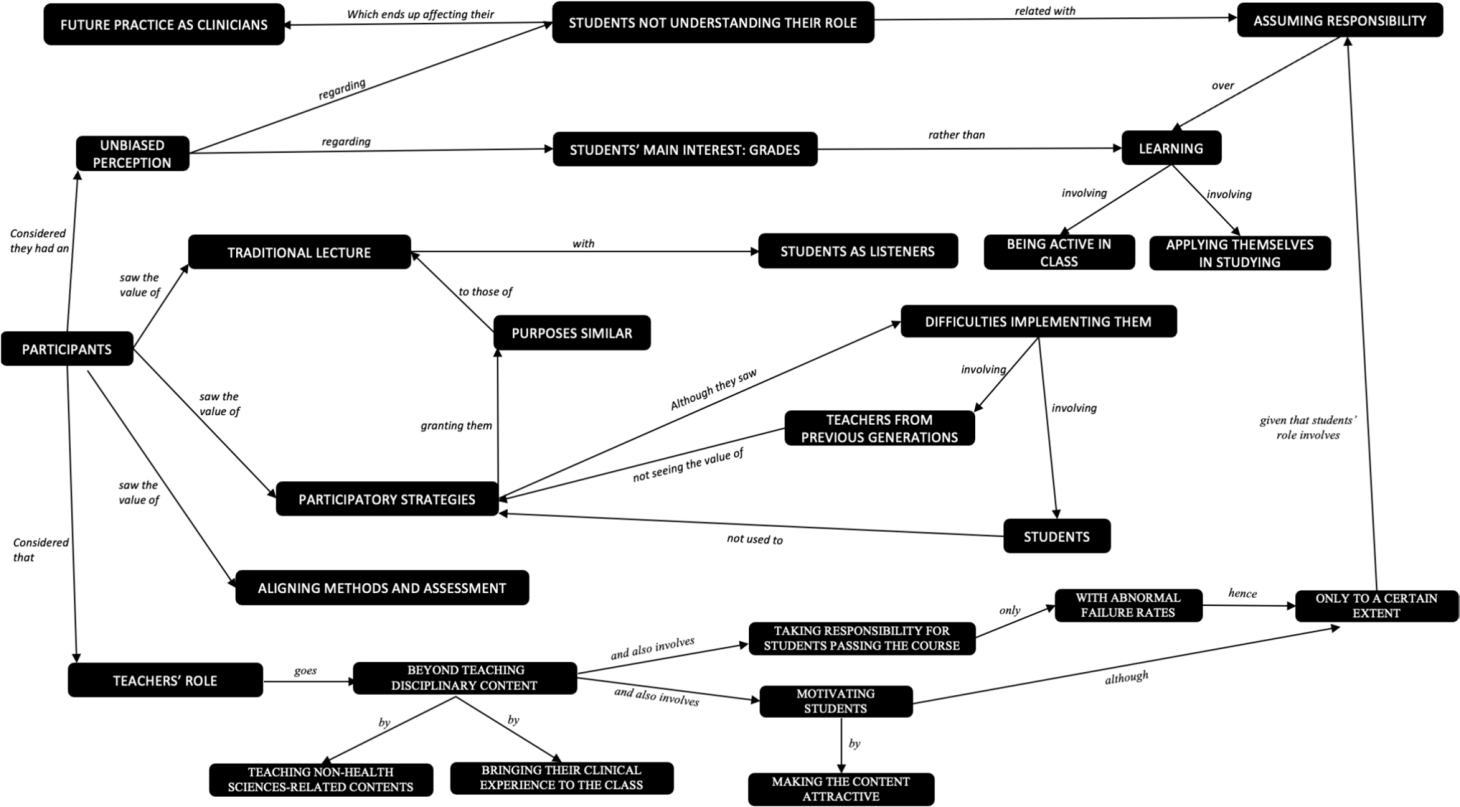
Viewpoints of the participants

Figure 1 reveals what transpires from the analysis of the participants’ conversations in regard to the two main analytical dimensions for this study: their ideas on teaching and learning methods and their viewpoints on the roles of teachers and learners in the teaching and learning process.

In the following lines, we summarize these results and exemplify them with excerpts from the conversations.

1. Perceptions about their role as teachers

The participants perceived that their role as teachers was related to:

(1.1) Going beyond teaching health sciences theory and concepts. As teachers, but also as clinical professionals, the participants thought that they could offer their students something beyond theoretical explanations:

I think I can provide them with many other things, not just explaining that a muscle goes from here to there (…). Muscles are something they need to learn by memorizing; I told them that I have no intention of explaining musculature in any class.

To that end, they reported that their role in class should move towards showing the students exactly what they could not find in the textbooks—reality and experiences:

There is no reason for me to explain what they already have in the book. What I can offer them in class time is my experience: If I were to find these complications, what would I do?

Likewise, they acknowledged that they should teach more than only health sciences–related content, although they found difficulties doing this:

Beyond teaching contents and theory about surgery, I always think that I need to try to teach them how to learn, but I don’t manage to do it.

(1.2) Motivating to a certain extent. The participants recognized that part of their role as teachers was to motivate their students. Their approach involved bringing the contents closer and making them attractive to the students:

You need to make it attractive; maybe for us it already is, but maybe it’s not like that for them.

Although the participants self-reflected on their responsibility in motivating their students, at the same time, they were reluctant to dedicate themselves excessively to motivating the students, connecting it to their educational philosophy. The following is an excerpt from an exchange on this issue:

- (…) It’s like if there is this excessive pressure to make them [the students] pay attention (…).

- These people from education, (…) they nearly suggest that you have to stand over a table to start dancing in order to make your students follow you. I can try to do my best, to be appealing, but if they follow, they follow, and if not….

(1.3) Being responsible for students’ passing the course to a certain extent. The participants thought that they were responsible when students failed a subject, but only when failures occurred at what they considered abnormal rates:

I’ve always thought that if a major group of students fails, part of the responsibility is mine. If 10 or 15 fail out of 80, that’s normal.

These thoughts about the passing/failing rates that the participants considered normal were discussed oftentimes, and we found examples of percentages that HSEs considered not only normal but satisfactory:

In my case, if it’s 70% of the students that are following me, I am more than happy with that (…). It could be that I’m too easily satisfied; it’s probably my age.

2. Perceptions about the students and their role as learners

Our participants perceived and discussed their students’ role as learners, arguing that their vision was unbiased:

(My perception) it’s not pessimistic; it’s realistic (…). I mean, the students are pragmatic; they need to attend to different demands, so they keep things uncomplicated.

They discussed the students’ role in the following terms:

(2.1) The learners’ role should be learning. Participants argued that students should focus on learning; however, they perceived that their students showed more interest in their grades (often, their primary goal) than in learning:

(…) what you should be proud of is of knowing well the content, that’s the goal; grades are separate matter.

(2.2) Learners should be responsible for their learning. Participants discussed that their students had a share of responsibility in their own learning that they needed to assume:

It’s like when you cook a dish and share responsibilities; we all have to do our part.

Part of this responsibility, according to the participants, should be exhibited in class by being more active, a situation related to what they consider is a lack of participation in their classes:

They found it difficult to participate (…). It’s not interesting for them. You try with different mechanisms to get to them, but….

Another side of that responsibility has to do with the students applying themselves in studying outside of class, even more so when they consider that classroom time is often not enough to work on all the contents of the syllabus. The following excerpt reflects this aspect:

- I can leave a class satisfied if I have one specific goal and they get to it, but the rest, all the volume of the contents, if they don’t work on them later at home, they will not get it (…).

- But their concept of “a lot of work” is distorted.

Overall, the idea of the students not taking their share of the responsibility was central to their discussions:

So, what does it mean? That they’re not reading it? It’s the same thing again (…). You have to assume that they’re not doing what they’re supposed to.

They connected this issue to the students’ future professional practice as clinicians:

To me, in health sciences education, it’s a failure that they don’t understand what their role is and how to fulfill it. I think that later this could be transferred to how they work in teams. I see that it happens.

3. Perceptions about teaching and learning methods

Participants discussed their perceptions about teaching-learning strategies in the following terms:

(3.1) On methods alternative to the lecture. Participants recognized the value of strategies other than the lecture for being more participatory:

If we use different methods more and more when we teach, I think that at some point we’ll begin to see changes [in the students’ attitudes].

However, they viewed these alternative strategies as having purposes like those of the lecture:

(…) this type of class [flipped classroom], when you finish, the students will have assimilated the same things to what they would in a regular lecture.

At the same time, participants recurrently shared comments connecting the difficulty of changing teaching practices to a generational gap among lecturers:

We can continue working in this way, but I insist that, until we have a generational shift [things will remain similar]. There are many professors who tell you [when discussing alternative teaching and learning methods]: "What are you talking about? Classes are like this, period."

(3.2) On the traditional lecture. Even if some of the participants’ discourse favored the use of alternatives to the traditional lecture, they persistently continued defending lectures as a valid way to contribute to the students’ learning:

This [traditional lecture] is more boring, but not everything can be joy!

Also, this defense of traditional lectures rested on the idea of students as listeners:

I continue educating myself and, honestly, as a student I appreciate them. I go to listen, and I appreciate it because I’m paying to listen to what they say, their experiences.

Concurrently, they also justified maintaining lectures because their students were not used to alternative strategies:

Students come from school lectures. As many changes as schools are trying to implement, it’s still like that; so, we suddenly set up this type of class and it overwhelms them because they’re not used to it.

(3.3) On aligning assessment and teaching strategies. Although the participants had different perspectives on how to approach this, they recognized the importance of aligning assessment and teaching strategies. The following excerpt illustrates this:

- Let’s start from the end. How do we assess? And, depending on how we want to assess, we will need to teach (…). The thing is: how do we want to assess? And from that, how do we want to teach?

- No. I would do it the other way around. I can think of a different [methodological] approach for the different lessons and, from that idea, assess each part differently.

- All right, but you’re already thinking about how to assess. They are tied; I agree.

## Discussion

Our results have shown how the participants’ viewpoints constitute a complex amalgam of educational ideas with the sole foundation of their teaching experience.

Despite the lack of educational training, in the participants’ discourse we identified different elements that evince how they were able to conduct meaningful pedagogical deliberations. One of these involves their discussion of the importance of aligning assessment and teaching methods (see 3.3 in the results), implicitly referring to the idea of “constructive alignment” [30], a key principle when approaching the design of teaching and learning experiences. Another example is how they envisioned the connection between being responsible and engaged in learning and students’ later professionalism as clinicians, a relation supported by the literature on the subject [31, 32]. Also, the participants’ discourse granted importance to the students’ personal responsibility for their learning, a component that is essential for metacognition and lifelong learning [33]. Lastly, the participants also valued teaching strategies to promote learning beyond the traditional lecture.

Still, for some of these elements, the participants’ perspective was rather limited. In this sense, while they recognized the importance of using alternatives to the traditional lecture, the lack of differentiation in the purposes of these strategies to those of a lecture and their arguments in favor of a lecture where students take the sole role of listeners—concurrently challenging their own notion that part of the students’ responsibility over their learning has to do with being active in class—evince a simplistic view of teaching and raise epistemological concerns that, without educational training, could hardly be addressed [11].

Likewise, their support of traditional lectures is not aligned with what current research [34, 35] tells us regarding the students’ preferences, showing that lectures appear among the least-preferred options of health sciences students and that, even if some students express concerns about alternative strategies, they usually show strong satisfaction with these and prefer them to instruction based on lectures. Research [36] in medical education showing students’ appreciation for traditional lectures reveals that this is merely circumscribed to descriptive contents (e.g., functional and descriptive anatomy), which are, precisely, the types of contents that our participants would rather not teach, as they consider their role to go beyond teaching theoretical contents that could be found in a textbook. In this sense, as recent research among medical educators suggests [37], by maintaining this point of view about traditional lectures, our participants seemed to be missing the actual learning needs of this generation of students.

The participants recognized difficulties in shifting towards more participatory teaching and learning strategies; however, the nature of these difficulties was always extrinsic, as it was others (students or older lecturers) who were reluctant or unaccustomed to these strategies, not themselves. We have already described how students’ preferences are not aligned with this idea; at the same time, while some factors with an impact on the successful implementation of active learning strategies could partly be of an extrinsic nature—students’ overall workload, their interest in the topic, their confidence, class size, etc. [38]—literature clearly evinces that these strategies’ effectiveness relies heavily on how they are put into practice [39] and on the lecturers’ educational competence, showing a positive correlation between advanced educational training and the understanding of active learning [40].

Another instance of this limited perspective when deliberating about educational topics emerges in how the participants recognized the students’ responsibility over their learning only in terms of being active in class and studying hard out of class, far from more developed understandings of responsibility and accountability [41] and not considering their own role in training students on how to be responsible, even more important given their concerns about their students’ approach to learning (unbiased, from their perspective). While the concerns they expressed are similar to those that we find in earlier research regarding the lack of responsibility and motivation of students [37], in order for students to attach importance to learning and to be motivated and responsible, teachers should show a clear commitment to the educational practice through their teaching approach and methods [7, 32]. In other words, the teachers’ role is key in contributing to the development of the students’ metacognitive skills regarding learning to learn and the self-regulation of learning [42].

Nevertheless, the participants’ vision of their role did not include such perspective related to the responsibility to train the students to fulfill their role as learners. The participants recognized that their role as teachers involved going beyond theoretical explanations of the content to bring their experience and clinical ways of doing to the class while also introducing non-health science contents that could be useful for health sciences professionals. With this, they implicitly identified themselves and intended to act as role models, a core competence for HSEs [13]. However, they approached their role as models from a teacher-centered perspective, acknowledging the importance of bringing their clinical expertise to the class but failing to contemplate what their students should do with it other than listening; differently, in a more student-centered approach, teachers would encourage students in critical assessment of and reflection on the model’s ways of doing [43].

The participants understood motivating students to be part of their role and, in doing so, they implicitly identified a key component of active learning for medical education: the design and use of strategies to promote students’ intentional engagement [11]. Nonetheless, their view of this responsibility was limited and, at the same time, exhibited an opposed attitude towards what they considered to be an educational trend overemphasizing the motivational role of teachers; overall, they evinced a simplified vision of the impact of motivation on students’ attitudes towards learning [44] and the key role of teachers and their pedagogical strategies—even more if they stimulate participation [45]—rather than lecturing in motivation and learning [46].

Lastly, the participants considered that their role involved being responsible for the students’ course results, but only when the percentage of course failures was higher than what they considered normal. This approach partly echoes recent research pointing out that, rather than instructors, it is students who have the greater control over their evaluations [47], but it also frames the discussion on the students’ outcomes in terms of grades (or passing/failing), rather than of learning, mirroring the approach they concurrently critiqued to their students. Yet another example of a challenging conception that, in this case, evinces a problematic approach when addressing the role and responsibility of lecturers over their students’ learning that previous studies in medical education have already suggested [6] and adds to the limited perception of their own educational role.

## Conclusions and Implications

On certain occasions, the lack of formal training can be compensated by accumulated experience. However, it becomes problematic when what should be exceptional turns into the norm, especially when this shift has implications for the learning process of health science professionals and, consequently, for their patients and society at large. This study demonstrates that, although educators without formal pedagogical training may independently formulate certain educational ideas that align with existing literature, the development and deliberation surrounding these ideas tend to oversimplify their meaning, lack proper foundations, and fail to acknowledge the necessary nuances and perspectives required for high-quality teaching practice, often approaching their role from a teacher-centered perspective. More concerning, perhaps, is that the study reveals a tendency among these educators to externalize challenges and exhibit inconsistencies that go unrecognized—traits uncharacteristic of a professional educator. These findings underscore the importance of pedagogical reflection and reinforce previous research [48] suggesting the need to grant greater value and centrality to pedagogical practice and training in health sciences education.

## Limitations and Future Research

Some limitations of this study should be highlighted. First, the sample size is relatively small and drawn from a particular context; studies with a bigger and more diverse sample might reveal different results. Nonetheless, in our analysis, we identified dimensions that are congruent with data from diverse settings. Second, our participants participated voluntarily in this study, and it is possible that study volunteers self-selected for their interest in teaching and learning. However, this limitation is found in most education research, since teachers who are not interested in teaching and learning usually do not open their educational practice to be observed. Also, we did not consider how the different career stages of our participants could have affected the conversations. Although future research could address this limitation, during our observations, we found that early-career teachers shared their ideas more than more experienced teachers, at least alleviating the concern that new teachers might feel too intimidated to participate. Finally, due to the collaborative process that our participants carried out to design, deliver, and discuss their lessons, their reported perceptions became intertwined; we therefore did not distinguish participants from the different health sciences fields in our analysis. Future research could also delve into the question of possible different perceptions among educators from different health disciplines.

## Data Availability

The data that support the findings of this study are available from the corresponding author (GH) upon reasonable request.
